# Supplementary material for: DBSCAN and GIE, Two Density-Based “Grid-Free” Methods for Finding Areas of Endemism: A Case Study of Flea Beetles (Coleoptera, Chrysomelidae) in the Afrotropical Region
Source: Insects. 2021 Dec 13;12(12):1115. doi: 10.3390/insects12121115 (PMC8708620; doi:10.3390/insects12121115)
Supplement: Supplementary file 1 [file insects-12-01115-s001.zip › insects-1490988/Supplementary_Material_File_S1_Supplementary References.pdf]

1. BIONDI M (1998) Revision of the Genus *Chirodica* Germar with description of five new species (Coleoptera Chrysomelidae). Proceedings of the Fourth International Symposium on the Chrysomelidae, XX I.C.E. Firenze, 1996. Mus. reg. Sci. nat. Torino, 1998: 17–48.
2. BIONDI M (1998) The genus *Malvernia* Jacoby with description of a new species from South Africa (Coleoptera, Chrysomelidae). Nouvelle Revue d'Entomologie (Nouvelle série.), 15 (1): 37–43.
3. BIONDI M (1999) The black *Longitarsus* species associated with Boraginaceae in South Africa (Coleoptera, Chrysomelidae, Alticinae). In: M. L. Cox (ed.), Advances in Chrysomelidae Biology, 1: 515–531.
4. BIONDI M (2000) *Biodontocnema* brunnea n.gen. and n.sp. from South Africa (Coleoptera: Chrysomelidae: Alticinae). The Coleopterists Bulletin, 54 (3): 347–350, doi: 10.1649/0010-065X(2000)054[0347:BBNGAN]2.0.CO;2
5. BIONDI M (2001) New flea beetle genus and species (Coleoptera: Chrysomelidae, Alticinae) from Central Africa. The Canadian Entomologist, 133: 643–649, doi: 10.4039/Ent133643-5
6. BIONDI M (2001) Revision of the *Chaetocnema* species from Madagascar. (Coleoptera, Chrysomelidae, Alticinae). European of Entomological Journal, 98: 233–248, doi: 10.14411/eje.2001.040
7. BIONDI M (2002) Comparative analysis of *Chaetocnema* Stephens and its kindred genera, with description of a new genus from the Indian Ocean (Seychelles) (Coleoptera, Chrysomelidae, Alticinae). Italian Journal of Zoology, 69: 22–33.
8. BIONDI M (2017) *Hesperoides*, a new "hairy" flea beetle genus from southern Africa (Coleoptera: Chrysomelidae, Galerucinae, Alticini). Fragmenta Entomologica, 49 (2):151–158, doi: 10.4081/fe.2017.257
9. BIONDI M, DE NARDIS G (2000) The *Chaetocnema* of the *longicornis* species-group: systematics and geographic distribution (Coleoptera: Chrysomelidae, Alticinae). Insects Systematics & Evolution, 32: 27–42, doi: 10.1163/187631200X00291
10. BIONDI M, D'ALESSANDRO P (2019) *Psylliodes shirensis*, a new replacement name for Psylliodes shira Biondi & D'Alessandro (Coleoptera: Chrysomelidae). Fragmenta entomologica, Short scientific note, 51 (1): 27. doi: 10.4081/fe.2019.340
11. BIONDI M, D'ALESSANDRO P (2003) *Drakensbergianella rudebecki*, new genus and new species from the mountains of the Southern Africa, and taxonomic observations on *Gabonia* Jacoby and related genera (Insecta, Coleoptera, Chrysomelidae, Alticinae). Zoologischer Anzeiger, 242: 97–106, doi: 10.1078/0044-5231-00090
12. BIONDI M, D'ALESSANDRO P (2004) Revision of the Afrotropical flea beetle genus *Collartaltica* Bechyné with description of two new species (Coleoptera, Chrysomelidae, Alticinae). Insect Systematics & Evolution, 35: 285–298, doi: 10.1163/187631204788920239
13. BIONDI M, D'ALESSANDRO P (2005) *Chaetocnema conducta* (Motschulsky) and its kindred species in the Afrotropical Region, with description of *C. lopatini*, a new species from Central Africa (Coleoptera, Chrysomelidae, Alticinae). In: Konstantinov A, Tishechkin A, Penev L (eds) Contributions to Systematics and Biology of Beetles. Papers celebrating the 80th birthday of Igor Konstantinovich Lopatin. p. 3 –16, Sofia: Pensoft Publishers, ISBN: 954-642-233-9
14. BIONDI M, D'ALESSANDRO P (2006) A revision of the South African *Chaetocnema gahani* species-group, with descriptions of four new flea beetle species (Coleoptera: Chrysomelidae). Annales de la Société Entomologique de France, 42(2): 183 –196
15. BIONDI M, D'ALESSANDRO P (2007) *Afroaltica subaptera*, new genus and new species of flea beetles from the southern Africa (Coleoptera: Chrysomelidae: Alticinae). European Journal of Entomology, 104: 99–103, doi: 10.14411/eje.2007.015
16. BIONDI M, D'ALESSANDRO P (2008) Revision of the *Chaetocnema pulla* species-group from the Afrotropical region with description of a new species from Central Africa (Coleoptera: Chrysomelidae). p. 265-285, in: Jolivet P, Santiago-Blay J, Schmitt M. (eds) Research on Chrysomelidae Volume I. Leiden: Brill, doi: 10.1163/9789047427858_007
17. BIONDI M, D'ALESSANDRO P (2008) Taxonomical revision of the *Longitarsus capensis* species-group: an example of Mediterranean-southern African disjunct distributions (Coleoptera: Chrysomelidae). European Journal of Entomology, 105: 719–736, doi: 10.14411/eje.2008.099

18. BIONDI M, D'ALESSANDRO P (2010) Revision of the Afrotropical flea beetle genus *Serraphula* Jacoby and description of *Bechynella*, a new genus from Western and Central Africa (Coleoptera: Chrysomelidae: Alticinae). Zootaxa, 2444: 1–44, doi: 10.5281/zenodo.195007
19. BIONDI M, D'ALESSANDRO P (2011) *Jacobyana* Maulik, an Oriental flea beetle genus new for the Afrotropical Region with description of three new species from Central and Southern Africa (Coleoptera, Chrysomelidae, Alticinae). Zookeys, 86: 47–59, doi: 10.3897/zookeys.86.804
20. BIONDI M, D'ALESSANDRO P (2013) *Ntaolaltica* and *Pseudophygasia*, two new flea beetle genera from Madagascar (Coleoptera: Chrysomelidae: Galerucinae: Alticini). Insect Systematics & Evolution, 44: 93–106, doi: 10.1163/1876312X-04401004
21. BIONDI M, D'ALESSANDRO P (2013) The genus *Chabria* Jacoby: First records in the Afrotropical region with description of three new species from Madagascar and annotated worldwide species catalogue (Coleoptera, Chrysomelidae, Galerucinae, Alticini). Zoologischer Anzeiger, 252: 88–100, doi: 10.1016/j.jcz.2012.03.005
22. BIONDI M, D'ALESSANDRO P (2015) Revision of the Afrotropical genus *Notomela* Jacoby, 1899 with description of N. joliveti sp. n. from Principe Island (Coleoptera, Chrysomelidae, Galerucinae, Alticini). Zookeys, 547: 63–74, doi: 10.3897/zookeys.547.9375
23. BIONDI M, D'ALESSANDRO P (2016) Revision of *Diphaulacosoma* Jacoby, an endemic flea beetle genus from Madagascar, with description of three new species (Coleoptera: Chrysomelidae, Galerucinae, Alticini). Fragmenta entomologica, 48: 14 –151, doi: 10.4081/fe.2016.181
24. BIONDI M, D'ALESSANDRO P (2017) *Guilielmia* Weise, a little known Afrotropical flea beetle genus: systematic affinities and description of a second new species from Central Africa (Coleoptera, Chrysomelidae, Galerucinae, Alticini). Zootaxa, 4323: 572–578, doi: doi.org/10.11646/zootaxa.4323.4.9
25. BIONDI M, D'ALESSANDRO P (2017) *Longitarsus doeberli*, a wingless new species from Socotra Island (Coleoptera: Chrysomelidae). Acta Entomologica Musei Nationalis Pragae, 57: 165–172, doi: 10.1515/aemnp-2017-0116
26. BIONDI M, D'ALESSANDRO P (2018) Taxonomic revision of the genus *Angulaphthona* (Coleoptera: Chrysomelidae: Galerucinae: Alticini). European Journal of Entomology, 115: 30–44, doi: 10.14411/eje.2018.005
27. BIONDI M, D'ALESSANDRO P (2018) Two new species of the flea beetle genus *Psylliodes* Latreille of the montana species-group from Eastern Africa (Coleoptera: Chrysomelidae). Fragmenta entomologica, 50(2): 87-94, doi: 10.4081/fe.2018.305
28. BIONDI M, D'ALESSANDRO P (2018). Two new species of *Chaetocnema* Stephens from South Africa (Coleoptera: Chrysomelidae, Galerucinae, Alticini). Fragmenta entomologica, 50(1): 11–18, doi: 10.4081/fe.2018.279
29. BIONDI M, IANNELLA M, D'ALESSANDRO P (2019) Unravelling the taxonomic assessment of an interesting new species from Socotra Island: *Blepharidina socotrana* sp. nov. (Coleoptera: Chrysomelidae). Acta Entomologica Musei Nationalis Pragae, 59(2): 499–505, doi: 10.2478/aemnp-2019-0040
30. BIONDI M, IANNELLA M, D'ALESSANDRO P (2020) *Adamastoraltica humicola*, new genus and new species: the first example of possible moss-inhabiting flea beetle genus from sub-Saharan Africa (Coleoptera, Chrysomelidae, Galerucinae) Zootaxa 4763(1): 099–108, doi: 10.11646/zootaxa.4763.1.8
31. BIONDI M, URBANI F, D'ALESSANDRO P (2013). Revision of the *Aphthona cookei* species group in Sub-Saharan Africa: pests of Jatropha curcas L. in biodiesel plantations (Coleoptera, Chrysomelidae, Galerucinae, Alticini). Entomologia, 1: 41–59, doi: 10.4081/entomologia.2013.e7
32. D'ALESSANDRO P, BIONDI M (2018) *Ugandaltica* gen. n., a tiny flea beetle from forest canopy in Central Africa (Coleoptera, Chrysomelidae, Galerucinae, Alticini). Zookeys vol. 746 p. 123–136, doi: 10.3897/zookeys.746.23637
33. D'ALESSANDRO P, FRASCA R, GROBBELAAR E, IANNELLA M, BIONDI M (2018) Systematics and biogeography of the Afrotropical flea beetle subgenus *Blepharidina* (Afroblepharida) Biondi & D'Alessandro, with description of seven new species (Coleoptera, Chrysomelidae, Galerucinae, Alticini). Insect Systematics & Evolution, 49: 443–480, doi: 10.1163/1876312X-00002182

34. D’ALESSANDRO P, IANNELLA M, BIONDI M (2019) Revision of the Afrotropical flea beetle subgenus *Blepharidina* s. str. Bechyné (Coleoptera, Chrysomelidae). Zootaxa: 4545 (1): 032–060, doi: 10.11646/zootaxa.4545.1.2
35. D’ALESSANDRO P, IANNELLA M, GROBBELAAR E, BIONDI M (2020) Revision of the *Calotheca nigrotessellata* species group from southern Africa, with description of two new species (Coleoptera: Chrysomelidae: Galerucinae: Alticini). Fragmenta entomologica 52 (2): 169–182, doi: 10.4081/fe.2020.457
36. D’ALESSANDRO P, IANNELLA M, GROBBELAAR E, BIONDI M (2021) Taxonomic revision of the *Calotheca parvula* species group from southern Africa, with descriptions of three new species (Coleoptera, Chrysomelidae). African Invertebrates 62(1): 315–337, doi: 10.3897/AfrInvertebr.62.62426
37. D'ALESSANDRO P, BIONDI M (2011) The Afrotropical genus *Afroaltica* Biondi & D’Alessandro (Coleoptera: Chrysomelidae: Alticinae): new data and description of a new species from Limpopo (Republic of South Africa). Annales de la Société Entomologique de France, 47: 365–370, doi: 10.1080/00379271.2011.10697730
38. D'ALESSANDRO P, GROBBELAAR E, BIONDI M (2012) Revision of the genus *Stegnaspea* Baly with descriptions of five new species from southern Africa (Coleoptera: Chrysomelidae: Galerucinae: Alticini). Insect Systematics & Evolution, 43: 11–33, doi: 10.1163/187631212X626032
39. D'ALESSANDRO P, URBANI F, BIONDI M (2014) Biodiversity and biogeography in Madagascar: revision of the endemic flea beetle genus *Neodera* Duvivier, 1891 with description of 19 new species (Coleoptera, Chrysomelidae, Galerucinae, Alticini). Systematic Entomology, 39(4): 710–748, doi: 10.1111/syen.12082
40. IANNELLA M, D’ALESSANDRO P, DE SIMONE W, BIONDI M (2021) Habitat Specificity, Host Plants and Areas of Endemism for the Genera-Group *Blepharida* s.l. in the Afrotropical Region (Coleoptera, Chrysomelidae, Galerucinae, Alticini). Insects, 12, 299: 1–16, doi: 10.3390/
