# Supplementary material for: DBSCAN and GIE, Two Density-Based “Grid-Free” Methods for Finding Areas of Endemism: A Case Study of Flea Beetles (Coleoptera, Chrysomelidae) in the Afrotropical Region
Source: Insects. 2021 Dec 13;12(12):1115. doi: 10.3390/insects12121115 (PMC8708620; doi:10.3390/insects12121115)
Supplement: Supplementary file 1 [file insects-12-01115-s001.zip › insects-1490988/Supplementary_Material_File_S3_List of sinendemisms in the AoEs.pdf]

List of the sinendemisms in each AoE identified by the DBSCAN and GIE methods

Density-based spatial clustering of applications with noise (DBSCAN)

100km (maximum diameter of distribution) 100km (maximum distance of centroids) 5syn (number of synendemisms)

Cluster WCP Western Cape Province 21

Adamastoraltica humicola
Chaetocnema adamastori
Chaetocnema chalcea
Chaetocnema saldanhai
Chaetocnema sp n 1
Chaetocnema sp n 8
Chaetocnema tablensis
Chirodica cedarbergensis
Longitarsus afromeridionalis
Longitarsus cedarbergensis
Longitarsus debiasei
Longitarsus hexrivierbergensis
Longitarsus luctuosus
Longitarsus lugubris
Longitarsus malherbei
Longitarsus neseri
Longitarsus piketbergensis
Longitarsus rouxi
Stegnaspea audisiana
Stegnaspea danielssoni
Stegnaspea penrithae

Cluster KAR Katanga region 6

Angulaphthona confusa
Chaetocnema muya
Chaetocnema reprehensa
Chaetocnema sp n 2
Chaetocnema sp n 3
Collartaltica nigrolucens

Cluster ANR Antananarivo region 10

Chabria betsimisaraka
Chabria bezanozana
Chaetocnema basipunctata
Chaetocnema cachani
Chaetocnema madascariensis
Neodera difficilis
Neodera diversitarsis
Neodera kraussi
Neodera pauliani0……….
Neodera straminea

Cluster MP-LI Mpumalanga-Limpopo 11

Afroaltica parvula
Chaetocnema mapumalangaensis
Chaetocnema sp n 4
Chaetocnema sp n 7
Chaetocnema sp n 9
Longitarsus transvaalensis
Serraphula alticola
Serraphula grobbelaariae
Serraphula mpumalangaensis
Serraphula transvaalensis
Serraphula wittmeri

Cluster DKM-KWN Drakensberg Mountains- KwaZulu-Natal 10

Chaetocnema barkeri
Chaetocnema mariobiondii
Chaetocnema sp n 5
Chaetocnema sp n 6
Drakensbergianella rudebecki
Serraphula audisiana
Serraphula colonnellii
Serraphula drakensbergensis
Serraphula natalensis
Serraphula osellai

Cluster BER	Betsiboka region	10

Diphaulacosoma crassicornis
Diphaulacosoma elegans
Diphaulacosoma nigroscutis
Neodera longicollis
Neodera micheli
Neodera reducta
Neodera sogai
Neodera vadoni
Pseudophygasia ambohitsitondroensis
Pseudophygasia apolinaria

Cluster AMM	Ambra Mountain	5

Diphaulacosoma bicolor
Neodera bernieri
Neodera breviantennata
Neodera fulva
Neodera opaca
Ntaolaltica antennata

300km 100km 5syn

Cluster WCP	Western Cape Province	32

Adamastoraltica humicola
Calotheca danielssoni
Calotheca pallida
Calotheca regularis
Chaetocnema adamastori
Chaetocnema audisiana
Chaetocnema chalcea
Chaetocnema saldanhai
Chaetocnema sp n 1
Chaetocnema sp n 8
Chaetocnema tablensis
Chirodica cedarbergensis
Chirodica chalcoptera unicolor
Chirodica outeniquensis
Longitarsus afromeridionalis
Longitarsus capensis
Longitarsus cedarbergensis
Longitarsus debiasei
Longitarsus hexrivierbergensis
Longitarsus luctuosus
Longitarsus lugubris
Longitarsus malherbei
Longitarsus melanicus
Longitarsus neseri
Longitarsus piketbergensis
Longitarsus rouxi
Longitarsus sudafricanus
Stegnaspea audisiana
Stegnaspea danielssoni
Stegnaspea debiasei
Stegnaspea endroedyi
Stegnaspea penrithae

Cluster KAR	Katanga region	6

Angulaphthona confusa
Chaetocnema muya
Chaetocnema reprehensa
Chaetocnema sp n 2
Chaetocnema sp n 3
Collartaltica nigrolucens

Cluster BER+ANR	Betsiboka region+Antananarivo region	35

Chabria betsimisaraka
Chabria bezanozana
Chaetocnema basipunctata
Chaetocnema cachani
Chaetocnema coronilla
Chaetocnema madascariensis
Diphaulacosoma crassicornis
Diphaulacosoma elegans
Diphaulacosoma jenisi
Diphaulacosoma nigroscutis
Neodera amplicollis
Neodera didiensis
Neodera difficilis
Neodera diversitarsis
Neodera emarginata
Neodera imitatrix
Neodera jenisi
Neodera kraussi
Neodera longicollis
Neodera madagassa

Neodera marojejyensis
Neodera micheli
Neodera pauliani
Neodera perroti
Neodera peyrierasi
Neodera picticornis
Neodera reducta
Neodera sogai
Neodera straminea
Neodera straminoides
Neodera vadoni
Neodera varicornis
Pseudophygasia ambohitsitondroensis
Pseudophygasia apolinaria
Pseudophygasia furax

Cluster DKM-KWN   Drakensberg Mountains-KwaZulu-Natal   13

Chaetocnema barkeri
Chaetocnema longicornis
Chaetocnema mariobiondii
Chaetocnema sp n 5
Chaetocnema sp n 6
Chaetocnema turneri
Chaetocnema zulu
Drakensbergianella rudebecki
Serraphula audisiana
Serraphula colonnellii
Serraphula drakensbergensis
Serraphula natalensis
Serraphula osellai

Cluster MP-LI   Mpumalanga-Limpopo   11

Afroaltica parvula
Chaetocnema mapumalangaensis
Chaetocnema sp n 4
Chaetocnema sp n 7
Longitarsus transvaalensis
Serraphula alticola
Serraphula duplessisi
Serraphula grobbelaariae
Serraphula mpumalangaensis
Serraphula transvaalensis
Serraphula wittmeri

Cluster AMM   Amber Mountain   6

Diphaulacosoma bicolor
Neodera bernieri
Neodera breviantennata
Neodera fulva
Neodera opaca
Ntaolaltica antennata

500km 100km 5syn
Cluster WCP   Western Cape Province   35

Adamastoraltica humicola
Calotheca danielssoni
Calotheca pallida
Calotheca regularis
Chaetocnema adamastori
Chaetocnema audisiana
Chaetocnema chalcea
Chaetocnema convexicollis
Chaetocnema saldanhai
Chaetocnema sp n 1
Chaetocnema sp n 8
Chaetocnema tablensis
Chirodica cedarbergensis
Chirodica chalcoptera unicolor
Chirodica fulvipes
CHirodica outeniquensis
Chirodica similfulva
Longitarsus afromeridionalis
Longitarsus capensis
Longitarsus cedarbergensis
Longitarsus debiasei
Longitarsus hexrivierbergensis
Longitarsus luctuosus
Longitarsus lugubris
Longitarsus malherbei
Longitarsus melanicus
Longitarsus neseri
Longitarsus piketbergensis
Longitarsus rouxi
Longitarsus sudafricanus
Stegnaspea audisiana
Stegnaspea danielssoni

Stegnaspea debiasei	*Stegnaspea penrithae*
Stegnaspea endroedyi	

Cluster KAR Katanga region 6

Angulaphthona confusa	*Chaetocnema sp n 2*
Chaetocnema muya	*Chaetocnema sp n 3*
Chaetocnema reprehensa	*Collartaltica nigrolucens*

Cluster BER+ANR Betsiboka region+Antananarivo region 40

Chabria betsimisaraka	*Neodera madagassa*
Chabria bezanozana	*Neodera marojejyensis*
Chaetocnema basipunctata	*Neodera micheli*
Chaetocnema cachani	*Neodera pauliani*
Chaetocnema coronilla	*Neodera perroti*
Chaetocnema madascariensis	*Neodera peyrierasi*
Chaetocnema orophila	*Neodera picticornis*
Diphaulacosoma crassicornis	*Neodera reducta*
Diphaulacosoma elegans	*Neodera similvadoni*
Diphaulacosoma jenisi	*Neodera sogai*
Diphaulacosoma nigroscutis	*Neodera straminea*
Neodera amplicollis	*Neodera straminoides*
Neodera didiensis	*Neodera transversicollis*
Neodera difficilis	*Neodera vadoni*
Neodera diversitarsis	*Neodera varicornis*
Neodera emarginata	*Pseudophygasia ambohitsitondroensis*
Neodera imitatrix	*Pseudophygasia analis*
Neodera jenisi	*Pseudophygasia apolinaria*
Neodera kraussi	*Pseudophygasia denotata*
Neodera longicollis	*Pseudophygasia furax*

Cluster DKM-KWN Drakensberg Mountains-KwaZulu-Natal 16

Chaetocnema barkeri	*Chaetocnema zulu*
Chaetocnema brincki	*Drakensbergianella rudebecki*
Chaetocnema longicornis	*Serraphula audisiana*
Chaetocnema mariobiondii	*Serraphula colonnellii*
Chaetocnema sp n 5	*Serraphula drakensbergensis*
Chaetocnema sp n 6	*Serraphula monticola*
Chaetocnema sp n 12	*Serraphula natalensis*
Chaetocnema turneri	*Serraphula osellai*

Cluster MP-LI Mpumalanga-Limpopo 11

Afroaltica parvula	*Serraphula alticola*
Chaetocnema mapumalangaensis	*Serraphula duplessisi*
Chaetocnema sp n 4	*Serraphula grobbelaariae*
Chaetocnema sp n 7	*Serraphula mpumalangaensis*
Chaetocnema sp n 9	*Serraphula wittmeri*
Longitarsus transvaalensis	

Cluster AMM Amber Mountain 6

Diphaulacosoma bicolor
Neodera bernieri
Neodera breviantennata
Neodera fulva
Neodera opaca
Ntaolaltica antennata

100km 150km 5syn

Cluster WCP Western Cape Province 25

Adamastoraltica humicola
Calotheca pallida
Chaetocnema adamastori
Chaetocnema chalcea
Chaetocnema saldanhai
Chaetocnema sp n 1
Chaetocnema sp n 8
Chaetocnema tablensis
Chirodica cedarbergensis
Chirodica chalcoptera unicolor
Longitarsus afromeridionalis
Longitarsus cedarbergensis
Longitarsus debiasei
Longitarsus hexrivierbergensis
Longitarsus luctuosus
Longitarsus lugubris
Longitarsus malherbei
Longitarsus melanicus
Longitarsus neseri
Longitarsus piketbergensis
Longitarsus rouxi
Stegnaspea audisiana
Stegnaspea danielssoni
Stegnaspea endroedyi
Stegnaspea penrithae

Cluster MP-LI Mpumalanga-Limpopo 14

Afroaltica parvula
Calotheca luteotessellata
Chaetocnema mapumalangaensis
Chaetocnema sp n 4
Chaetocnema sp n 7
Chaetocnema sp n 9
CHaetocnema sp n 14
Longitarsus transvaalensis
Serraphula alticola
Serraphula duplessisi
Serraphula grobbelaariae
Serraphula mpumalangaensis
Serraphula transvaalensis
Serraphula wittmeri

Cluster KAR Katanga region 6

Angulaphthona confusa
Chaetocnema muya
Chaetocnema reprehensa
Chaetocnema sp n 2
Chaetocnema sp n 3
Collartaltica nigrolucens

Cluster KIL Kilimandjaro region 8

Blepharidina kasigauensis
Blepharidina macarthuri
Blepharidina ornaticollis
Calotheca jacobyi
Chaetocnema sp n 10
Chaetocnema sp n 11
Psylliodes montana
Psylliodes shirensis

Cluster BER+ANR Betsiboka region+Antananarivo region 25

Chabria betsimisaraka
Chabria bezanozana
Chaetocnema basipunctata
Chaetocnema cachani
Chaetocnema madascariensis
Diphaulacosoma crassicornis
Diphaulacosoma elegans
Diphaulacosoma nigroscutis
Neodera amplicollis
Neodera difficilis
Neodera diversitarsis
Neodera kraussi

Neodera longicollis
Neodera madagassa
Neodera marojejyensis
Neodera micheli
Neodera pauliani
Neodera perroti
Neodera reducta
Neodera sogai
Neodera straminea
Neodera vadoni
Pseudophygasia ambohitsitondroensis
Pseudophygasia apolinaria
Pseudophygasia furax

Cluster DKM-KWN Drakensberg Mountains - KwaZulu-Natal 13

Chaetocnema barkeri
Chaetocnema mariobiondii
Chaetocnema sp n 5
Chaetocnema sp n 6
Drakensbergianella rudebecki
Jacobyana sudafricana
Serraphula audisiana
Serraphula colonnellii
Serraphula drakensbergensis
Serraphula monticola
Serraphula natalensis
Serraphula osellai
Serraphula puncticollis

Cluster KLR Kivu Lake region 5

Chaetocnema rutovuensis
Chaetocnema varicornis
Guilielmia monticola
Guilielmia leleupi
Jacobyana centrafricana

Cluster AMM Amber Mountain 6

Diphaulacosoma bicolor
Neodera bernieri
Neodera breviantennata
Neodera fulva
Neodera opaca
Ntaolaltica antennata

300km 150km 5syn
Cluster WCP Western Cape Province 32

Adamastoraltica humicola
Calotheca danielssoni
Calotheca pallida
Calotheca regularis
Chaetocnema adamastori
Chaetocnema audisiana
Chaetocnema chalcea
Chaetocnema saldanhai
Chaetocnema sp n 1
Chaetocnema sp n 8
Chaetocnema tablensis
Chirodica cedarbergensis
Chirodica chalcoptera unicolor
Chirodica outeniquensis
Longitarsus afromeridionalis
Longitarsus capensis
Longitarsus cedarbergensis
Longitarsus debiasei
Longitarsus hexrivierbergensis
Longitarsus luctuosus
Longitarsus lugubris
Longitarsus malherbei
Longitarsus melanicus
Longitarsus neseri
Longitarsus piketbergensis
Longitarsus rouxi
Longitarsus sudafricanus
Stegnaspea audisiana
Stegnaspea danielssoni
Stegnaspea debiasei
Stegnaspea endroedyi
Stegnaspea penrithae

Cluster MP-LI Mpumalanga-Limpopo 14

Afroaltica parvula
Calotheca luteotessellata

Chaetocnema mapumalangaensis
Chaetocnema sp n 4
Chaetocnema sp n 7
Chaetocnema sp n 9
Chaetocnema sp n 14
Longitarsus transvaalensis
Serraphula alticola
Serraphula duplessisi
Serraphula grobbelaariae
Serraphula mpumalangaensis
Serraphula transvaalensis
Serraphula wittmeri

Cluster KAR Katanga region 6

Angulaphthona confusa
Chaetocnema muya
Chaetocnema reprehensa
Chaetocnema sp n 2
Chaetocnema sp n 3
Collartaltica nigrolucens

Cluster KIL Kilimandjaro region 11

Blepharidina kasigauensis
Blepharidina keniana
Blepharidina macarthuri
Blepharidina ornaticollis
Blepharidina regalini
Calotheca jacobyi
Chaetocnema sp n 10
Chaetocnema sp n 11
Collartaltica alluadi
Psylliodes montana
Psylliodes shirensis

Cluster BER+ANR Betsiboka region+Antananarivo region 35

Chabria betsimisaraka
Chabria bezanozana
Chaetocnema basipunctata
Chaetocnema cachani
Chaetocnema coronilla
Chaetocnema madascariensis
Diphaulacosoma crassicornis
Diphaulacosoma elegans
Diphaulacosoma jenisi
Diphaulacosoma nigroscutis
Neodera amplicollis
Neodera didiensis
Neodera difficilis
Neodera diversitarsis
Neodera emarginata
Neodera imitatrix
Neodera jenisi
Neodera kraussi
Neodera longicollis
Neodera madagassa
Neodera marojejyensis
Neodera micheli
Neodera pauliani
Neodera perroti
Neodera peyrierasi
Neodera picticornis
Neodera reducta
Neodera sogai
Neodera straminea
Neodera straminoides
Neodera vadoni
Neodera varicornis
Pseudophygasia ambohitsitondroensis
Pseudophygasia apolinaria
Pseudophygasia furax

Cluster DEK- KWN Drakensberg Mountains- KwaZulu-Natal 17

Chaetocnema barkeri
Chaetocnema longicornis
Chaetocnema mariobiondii
Chaetocnema sp n 5
Chaetocnema sp n 6
Chaetocnema turneri
Chaetocnema zulu
Drakensbergianella rudebecki
Jacobyana sudafricana
Longitarsus grobbelaariae
Serraphula audisiana
Serraphula colonnellii
Serraphula drakensbergensis
Serraphula monticola
Serraphula natalensis
Serraphula osellai

Serraphula puncticollis

Cluster KLR Kivu Lake region 5

Chaetocnema rutovuensis
Chaetocnema varicornis
Guilielmia leleupi
Guilielmia monticola
Jacobyana centrafricana

Cluster AMM Amber Mountain 6

Diphaulacosoma bicolor
Neodera bernieri
Neodera breviantennata
Neodera fulva
Neodera opaca
Ntaolaltica antennata

500km 150km 5syn
Cluster WCP Western Cape Province 35

Adamastoraltica humicola
Calotheca danielssoni
Calotheca pallida
Calotheca regularis
Chaetocnema adamastori
Chaetocnema audisiana
Chaetocnema chalcea
Chaetocnema convexicollis
Chaetocnema saldanhai
Chaetocnema sp n 1
Chaetocnema sp n 8
Chaetocnema tablensis
Chirodica cedarbergensis
Chirodica chalcoptera unicolor
Chirodica fulvipes
Chirodica outeniquensis
Chirodica similfulva
Longitarsus afromeridionalis
Longitarsus capensis
Longitarsus cedarbergensis
Longitarsus debiasei
Longitarsus hexrivierbergensis
Longitarsus luctuosus
Longitarsus lugubris
Longitarsus malherbei
Longitarsus melanicus
Longitarsus neseri
Longitarsus piketbergensis
Longitarsus rouxi
Longitarsus sudafricanus
Stegnaspea audisiana
Stegnaspea danielssoni
Stegnaspea debiasei
Stegnaspea endroedyi
Stegnaspea penrithae

Cluster MP-LI+DKM- KWN Mpumalanga-Limpopo+Drakensberg Mountains- KwaZulu-Natal 38

Afroaltica parvula
Calotheca carolineae
Calotheca luteotessellata
Calotheca wanati
Chaetocnema barkeri
Chaetocnema brincki
Chaetocnema frereensis
Chaetocnema longicornis
Chaetocnema mapumalangaensis
Chaetocnema mariobiondii
Chaetocnema phuthaditjhabensis
Chaetocnema sp n 4
Chaetocnema sp n 5
Chaetocnema sp n 6
Chaetocnema sp n 7
Chaetocnema sp n 9
Chaetocnema sp n 12
Chaetocnema sp n 14
Chaetocnema zulu
Drakensbergianella rudebecki
Jacobyana sudafricana
Longitarsus grobbelaariae
Longitarsus transvaalensis
Serraphula alticola
Serraphula audisiana
Serraphula burlischi
Serraphula colonnellii
Serraphula drakensbergensis
Serraphula duplessisi
Serraphula grobbelaariae

Serraphula monticola
Serraphula mpumalangaensis
Serraphula natalensis
Serraphula osellai
Serraphula puncticollis
Serraphula transvaalensis
Serraphula uysi
Serraphula wittmeri

Cluster KAR	Katanga region	6

Angulaphthona confusa
Chaetocnema muya
Chaetocnema reprehensa
Chaetocnema sp n 2
Chaetocnema sp n 3
Collartaltica nigrolucens

Cluster KIL	Kilimandjaro region	17

Blepharidina kasigauensis
Blepharidina kenyana
Blepharidina knighti
Blepharidina macarthuri
Blepharidina ornaticollis
Blepharidina regalini
Blepharidina scripta
Calotheca jacobyi
Calotheca sjostedti
Chaetocnema sp n 10
Chaetocnema sp n 11
Collartaltica alluadi
Psylliodes afromontana
Psylliodes kikuyana
Psylliodes manobioides
Psylliodes montana
Psylliodes shirensis

Cluster BER+ANR	Betsiboka region+Antananarivo region	40

Chabria betsimisaraka
Chabria bezanozana
Chaetocnema basipunctata
Chaetocnema cachani
Chaetocnema coronilla
Chaetocnema madascariensis
Chaetocnema orophila
Diphaulacosoma crassicornis
Diphaulacosoma elegans
Diphaulacosoma jenisi
Diphaulacosoma nigroscutis
Neodera amplicollis
Neodera didiensis
Neodera difficilis
Neodera diversitarsis
Neodera emarginata
Neodera imitatrix
Neodera jenisi
Neodera kraussi
Neodera longicollis
Neodera madagassa
Neodera marojejyensis
Neodera micheli
Neodera pauliani
Neodera perroti
Neodera peyrierasi
Neodera picticornis
Neodera reducta
Neodera similvadoni
Neodera sogai
Neodera straminea
Neodera straminoides
Neodera transversicollis
Neodera vadoni
Neodera varicornis
Pseudophygasia ambohitsitondroensis
Pseudophygasia analis
Pseudophygasia apolinaria
Pseudophygasia denotata
Pseudophygasia furax

Cluster KLR	Kivu Lake region	6

Chaetocnema impressicollis
Chaetocnema rutovuensis
Chaetocnema varicornis
Guilielmia leleupi
Guilielmia monticola
Jacobyana centrafricana

Cluster AMM	Amber Mountain	6

Diphaulacosoma bicolor
Neodera bernieri
Neodera breviantennata
Neodera fulva
Neodera opaca
Ntaolaltica antennata

100km 200km 5syn

Cluster WCP   Western Cape Province   25

Adamastoraltica humicola
Calotheca pallida
Chaetocnema adamastori
Chaetocnema chalcea
Chaetocnema saldanhai
Chaetocnema sp n 1
Chaetocnema sp n 8
Chaetocnema tablensis
Chirodica cedarbergensis
Chirodica chalcoptera unicolor
Longitarsus afromeridionalis
Longitarsus cedarbergensis
Longitarsus debiasei
Longitarsus hexrivierbergensis
Longitarsus luctuosus
Longitarsus lugubris
Longitarsus malherbei
Longitarsus melanicus
Longitarsus neseri
Longitarsus piketbergensis
Longitarsus rouxi
Stegnaspea audisiana
Stegnaspea danielssoni
Stegnaspea endroedyi
Stegnaspea penrithae

Cluster MP-LI+DKM- KWN   Mpumalanga-Limpopo+Drakensberg Mountains- KwaZulu-Natal   34

Afroaltica parvula
Calotheca carolineae
Calotheca luteotessellata
Calotheca wanati
Chaetocnema barkeri
Chaetocnema mapumalangaensis
Chaetocnema mariobiondii
Chaetocnema sp n 4
Chaetocnema sp n 5
Chaetocnema sp n 6
Chaetocnema sp n 7
Chaetocnema sp n 9
Chaetocnema sp n 14
Chaetocnema sudafricana
Drakensbergianella rudebecki
Jacobyana sudafricana
Longitarsus grobbelaariae
Longitarsus transvaalensis
Serraphula alticola
Serraphula audisiana
Serraphula burlischi
Serraphula colonnellii
Serraphula debiasei
Serraphula drakensbergensis
Serraphula duplessisi
Serraphula grobbelaariae
Serraphula monticola
Serraphula mpumalangaensis
Serraphula natalensis
Serraphula osellai
Serraphula puncticollis
Serraphula transvaalensis
Serraphula uysi
Serraphula wittmeri

Cluster KAR   Katanga region   7

Angulaphthona confuse
Chaetocnema kapirensis
Chaetocnema muya
Chaetocnema reprehensa
Chaetocnema sp n 2
Chaetocnema sp n 3
Collartaltica nigrolucens

Cluster KIL   Kilimandjaro region   9

Blepharidina kasigauensis
Blepharidina knighti
Blepharidina macarthuri
Calotheca jacobyi

Calotheca ornaticollis
Chaetocnema sp n 11
Chaetocnema sp n 15
Psylliodes montana
Psylliodes shirensis

Cluster BER+ANR	Betsiboka region+Antananarivo region	25

Chabria betsimisaraka
Chabria bezanozana
Chaetocnema basipunctata
Chaetocnema cachani
Chaetocnema madascariensis
Diphaulacosoma crassicornis
Diphaulacosoma elegans
Diphaulacosoma nigroscutis
Neodera amplicollis
Neodera difficilis
Neodera diversitarsis
Neodera kraussi
Neodera longicollis
Neodera madagassa
Neodera marojejyensis
Neodera micheli
Neodera pauliani
Neodera perroti
Neodera reducta
Neodera sogai
Neodera straminea
Neodera vadoni
Pseudophygasia ambohitsitondroensis
Pseudophygasia apolinaria
Pseudophygasia furax

Cluster KLR	Kivu Lake region	5

Chaetocnema rutovuensis
Chaetocnema varicornis
Guilielmia leleupi
Guilielmia monticola
Jacobyana centrafricana

Cluster AMM	Amber Mountain	6

Diphaulacosoma bicolor
Neodera bernieri
Neodera breviantennata
Neodera fulva
Neodera opaca
Ntaolaltica antennata

300km 200km 5syn

Cluster WCP	Western Cape Province	32

Adamastoraltica humicola
Calotheca danielssoni
Calotheca pallida
Calotheca regularis
Chaetocnema adamastori
Chaetocnema audisiana
Chaetocnema chalcea
Chaetocnema saldanhai
Chaetocnema sp n 1
Chaetocnema sp n 8
Chaetocnema tablensis
Chirodica cedarbergensis
Chirodica chalcoptera unicolor
Chirodica outeniquensis
Longitarsus afromeridionalis
Longitarsus capensis
Longitarsus cedarbergensis
Longitarsus debiasei
Longitarsus hexrivierbergensis
Longitarsus luctuosus
Longitarsus lugubris
Longitarsus malherbei
Longitarsus melanicus
Longitarsus neseri
Longitarsus piketbergensis
Longitarsus rouxi
Longitarsus sudafricanus
Stegnaspea audisiana
Stegnaspea danielssoni
Stegnaspea debiasei
Stegnaspea endroedyi
Stegnaspea penrithae

Cluster MP-LI+DKM- KWN	Mpumalanga-Limpopo+Drakensberg Mountains- KwaZulu-Natal	37

Afroaltica parvula
Calotheca carolineae
Calotheca luteotessellata
Calotheca wanati
Chaetocnema barkeri
Chaetocnema longicornis
Chaetocnema mapumalangaensis
Chaetocnema mariobiondii
Chaetocnema sp n 4
Chaetocnema sp n 5
Chaetocnema sp n 6
Chaetocnema sp n 7
Chaetocnema sp n 9
Chaetocnema sp n 14
Chaetocnema sudafricana
Chaetocnema turneri
Chaetocnema zulu
Drakensbergianella rudebecki
Jacobyana sudafricana
Longitarsus grobbelaariae
Longitarsus transvaalensis
Serraphula alticola
Serraphula audisiana
Serraphula burlischi
Serraphula colonnellii
Serraphula debiasei
Serraphula drakensbergensis
Serraphula duplessisi
Serraphula grobbelaariae
Serraphula monticola
Serraphula mpumalangaensis
Serraphula natalensis
Serraphula osellai
Serraphula puncticollis
Serraphula transvaalensis
Serraphula uysi
Serraphula wittmeri

Cluster KAR Katanga region 7

Angulaphthona confusa
Chaetocnema kapirensis
Chaetocnema muya
Chaetocnema reprehensa
Chaetocnema sp n 2
Chaetocnema sp n 3
Collartaltica nigrolucens

Cluster KIL Kilimandjaro region 15

Blepharidina kasigauensis
Blepharidina kenyana
Blepharidina knighti
Blepharidina macarthuri
Blepharidina ornaticollis
Blepharidina regalini
Calotheca jacobyi
Chaetocnema sp n 11
Chaetocnema sp n 15
Collartaltica alluadi
Psylliodes afromontana
Psylliodes kikuyana
Psylliodes manobioides
Psylliodes montana
Psylliodes shirensis

Cluster BER+ANR Betsiboka region+Antananarivo region 35

Chabria betsimisaraka
Chabria bezanozana
Chaetocnema basipunctata
Chaetocnema cachani
Chaetocnema coronilla
Chaetocnema madascariensis
Diphaulacosoma crassicornis
Diphaulacosoma elegans
Diphaulacosoma jenisi
Diphaulacosoma nigroscutis
Neodera amplicollis
Neodera didiensis
Neodera difficilis
Neodera diversitarsis
Neodera emarginata
Neodera imitatrix
Neodera jenisi
Neodera kraussi
Neodera longicollis
Neodera madagassa
Nisotra maiojejyensis
Neodera micheli
Neodera pauliani
Neodera perroti

Neodera peyrierasi
Neodera picticornis
Neodera reducta
Neodera sogai
Neodera straminea
Neodera straminoides
Neodera vadoni
Neodera varicornis
Pseudophygasia ambohitsitondroensis
Pseudophygasia apolinaria
Pseudophygasia furax

Cluster KLR Kivu Lake region 7

Chaetocnema impressicollis
Chaetocnema rutovuensis
CHaetocnema vanschuytbroecki
Chaetocnema varicornis
Guilielmia leleupi
Guilielmia monticola
Jacobyana centrafricana

Cluster AMM Amber Mountain 6

Diphaulacosoma bicolor
Neodera bernieri
Neodera breviantennata
Neodera fulva
Neodera opaca
Ntaolaltica antennata

500km 200km 5syn
Cluster WCP Western Cape Province 35

Adamastoraltica humicola
Calotheca danielssoni
Calotheca pallida
Calotheca regularis
Chaetocnema adamastori
Chaetocnema audisiana
Chaetocnema chalcea
Chaetocnema convexicollis
Chaetocnema saldanhai
Chaetocnema sp n 1
Chaetocnema sp n 8
Chaetocnema tablensis
Chirodica cedarbergensis
Chirodica chalcoptera unicolor
Chirodica fulvipes
Chirodica outeniquensis
Chirodica similfulva
Longitarsus afromeridionalis
Longitarsus capensis
Longitarsus cedarbergensis
Longitarsus debiasei
Longitarsus hexrivierbergensis
Longitarsus luctuosus
Longitarsus lugubris
Longitarsus malherbei
Longitarsus melanicus
Longitarsus neseri
Longitarsus piketbergensis
Longitarsus rouxi
Longitarsus sudafricanus
Stegnaspea audisiana
Stegnaspea danielssoni
Stegnaspea debiasei
Stegnaspea endroedyi
Stegnaspea penrithae

Cluster MP-LI+DKM- KWN Mpumalanga-Limpopo+Drakensberg Mountains- KwaZulu-Natal 41

Afroaltica parvula
Calotheca carolineae
Calotheca luteotessellata
Calotheca wanati
Chaetocnema barkeri
Chaetocnema brincki
Chaetocnema frereensis
Chaetocnema longicornis
Chaetocnema mapumalangaensis
Chaetocnema mariobiondii
Chaetocnema phuthaditjhabensis
Chaetocnema sp n 4
Chaetocnema sp n 5
Chaetocnema sp n 6
Chaetocnema sp n 7
Chaetocnema sp n 9
Chaetocnema sp n 12
Chaetocnema sp n 14

Chaetocnema sudafricana
Chaetocnema turneri
Chaetocnema zulu
Drakensbergianella rudebecki
Jacobyana sudafricana
Longitarsus grobbelaariae
Longitarsus transvaalensis
Serraphula alticola
Serraphula audisiana
Serraphula burlischi
Serraphula colonnellii
Serraphula debiasei
Serraphula drakensbergensis
Serraphula duplessisi
Serraphula grobbelaariae
Serraphula monticola
Serraphula mpumalangaensis
Serraphula natalensis
Serraphula osellai
Serraphula puncticollis
Serraphula transvaalensis
Serraphula uysi
Serraphula wittmeri

Cluster KAR Katanga region 7

Angulaphthona confusa
Chaetocnema kapirensis
Chaetocnema muya
Chaetocnema reprehensa
Chaetocnema sp n 2
Chaetocnema sp n 3
Collartaltica nigrolucens

Cluster KIL Kilimandjaro region 17

Blepharidina kasigauensis
Blepharidina kenyana
Blepharidina knighti
Blepharidina macarthuri
Blepharidina ornaticollis
Blepharidina regalini
Blepharidina scripta
Calotheca jacobyi
Calotheca sjostedti
Chaetocnema sp n 10
Chaetocnema sp n 11
Collartaltica alluadi
Psylliodes afromontana
Psylliodes kikuyana
Psylliodes manobioides
Psylliodes montana
Psylliodes shirensis

Cluster BER+ANR Betsiboka region+Antananarivo region 40

Chabria betsimisaraka
Chabria bezanozana
Chaetocnema basipunctata
Chaetocnema cachani
Chaetocnema coronilla
Chaetocnema madascariensis
Chaetocnema orophila
Diphaulacosoma crassicornis
Diphaulacosoma elegans
Diphaulacosoma jenisi
Diphaulacosoma nigroscutis
Neodera amplicollis
Neodera didiensis
Neodera difficilis
Neodera diversitarsis
Neodera emarginata
Neodera imitatrix
Neodera jenisi
Neodera kraussi
Neodera longicollis
Neodera madagassa
Neodera marojejyensis
Neodera micheli
Neodera pauliani
Neodera perroti
Neodera peyrierasi
Neodera picticornis
Neodera reducta
Neodera similvadoni
Neodera sogai
Neodera straminea
Neodera straminoides
Neodera transversicollis
Neodera vadoni
Neodera varicornis
Pseudophygasia ambohitsitondroensis
Pseudophygasia analis
Pseudophygasia apolinaria

Pseudophygasia denotata	*Pseudophygasia furax*

Cluster KLR	Kivu Lake region	7

Chaetocnema impressicollis	*Guilielmia leleupi*
Chaetocnema rutovuensis	*Guilielmia monticola*
Chaetocnema vanshuytbroecki	*Jacobyana centrafricana*
Chaetocnema varicornis	

Cluster AMM	Amber Mountain	6

Diphaulacosoma bicolor	*Neodera fulva*
Neodera bernieri	*Neodera opaca*
Neodera breviantennata	*Ntaolaltica antennata*

Geographical Interpolation of Endemism

Class 1: 100 km (Maximum distance between centroids)

KAR Katanga region 6

28,"Angulaphthona confusa"
28,"Chaetocnema muya"
28,"Chaetocnema reprehensa"
28,"Chaetocnema sp n 2"
28,"Chaetocnema sp n 3"
28,"Collartaltica nigrolucens"

AMM Amber Mountain 6

32,"Diphaulacosoma bicolor "
32,"Neodera bernieri"
32,"Neodera breviantennata"
32,"Neodera fulva"
32,"Neodera opaca"
32,"Ntaolaltica antennata"

BER+ANR Betsiboka region-Antananarivo region 28

33,"Chabria betsimisaraka"
33,"Chabria bezanozana"
33,"Chaetocnema basipunctata"
33,"Chaetocnema cachani"
33,"Chaetocnema coronilla"
33,"Chaetocnema madascariensis"
33,"Diphaulacosoma crassicornis"
33,"Diphaulacosoma elegans"
33,"Diphaulacosoma nigroscutis"
33,"Neodera amplicollis"
33,"Neodera difficilis"
33,"Neodera diversitarsis"
33,"Neodera jenisi"
33,"Neodera kraussi"
33,"Neodera longicollis"
33,"Neodera madagassa"
33,"Neodera marojejyensis"
33,"Neodera micheli"
33,"Neodera pauliani"
33,"Neodera reducta"
33,"Neodera similvadoni"
33,"Neodera sogai"
33,"Neodera straminea"
33,"Neodera straminoides"
33,"Neodera vadoni"
33,"Neodera varicornis"
33,"Pseudophygasia apolinaria"
33,"Pseudophygasia furax "

MP-LI+DKM-KWN Mpumalanga-Limpopo 13

47,"Afroaltica parvula"
47,"Calotheca luteotessellata"
47,"Chaetocnema mapumalangaensis "
47,"Chaetocnema sp n 4"
47,"Chaetocnema sp n 7"
47,"Chaetocnema sp n 9"
47,"Longitarsus transvaalensis"
47,"Serraphula alticola"
47,"Serraphula duplessisi"
47,"Serraphula grobbelaariae"
47,"Serraphula mpumalangaensis"
47,"Serraphula transvaalensis"
47,"Serraphula wittmeri"

WCP Western Cape Province 20

61,"Adamastoraltica humicola"
61,"Chaetocnema adamastori"
61,"Chaetocnema audisiana"
61,"Chaetocnema saldanhai"
61,"Chaetocnema sp n 1
61,"Chaetocnema tablensis"
61,"Chirodica cedarbergensis"
61,"Longitarsus afromeridionalis"
61,"Longitarsus capensis"
61,"Longitarsus cedarbergensis"
61,"Longitarsus debiasei"
61,"Longitarsus hexrivierbergensis"
61,"Longitarsus luctuosus"
61,"Longitarsus malherbei"
61,"Longitarsus neseri"
61,"Longitarsus piketbergensis"
61,"Longitarsus rouxi"
61,"Stegnaspea audisiana"

61,"Stegnaspea danielssoni"
61,"Stegnaspea penrithae"

Class 2: 150 km

KLR	Kivu Lake region	5

20,"Chaetocnema rutovuensis"
20,"Chaetocnema varicornis"
20,"Guilielmia leleupi"
20,"Guilielmia monticola"
20,"Jacobyana centrafricana"

KAR	Katanga region	6

29,"Angulaphthona confusa"
29,"Chaetocnema muya"
29,"Chaetocnema reprehensa"
29,"Chaetocnema sp n 2"
29,"Chaetocnema sp n 3"
29,"Collartaltica nigrolucens"

AMM	Amber Mountain	6

33,"Diphaulacosoma bicolor"
33,"Neodera bernieri"
33,"Neodera breviantennata"
33,"Neodera fulva"
33,"Neodera opaca"
33,"Ntaolaltica antennata"

BER+ANR	Betsiboka region-Antananarivo region	35

35,"Chabria betsimisaraka"
35,"Chabria bezanozana"
35,"Chaetocnema basipunctata"
35,"Chaetocnema cachani"
35,"Chaetocnema coronilla"
35,"Chaetocnema madascariensis"
35,"Diphaulacosoma crassicornis"
35,"Diphaulacosoma elegans"
35,"Diphaulacosoma jenisi"
35,"Diphaulacosoma nigroscutis"
35,"Neodera amplicollis"
35,"Neodera didiensis"
35,"Neodera difficilis"
35,"Neodera diversitarsis"
35,"Neodera emarginata"
35,"Neodera imitatrix"
35,"Neodera jenisi"
35,"Neodera kraussi"
35,"Neodera longicollis"
35,"Neodera madagassa"
35,"Neodera marojejyensis"
35,"Neodera micheli"
35,"Neodera pauliani"
35,"Neodera perroti"
35,"Neodera picticornis"
35,"Neodera reducta"
35,"Neodera similvadoni"
35,"Neodera sogai"
35,"Neodera straminea"
35,"Neodera straminoides"
35,"Neodera vadoni"
35,"Neodera varicornis"
35,"Pseudophygasia ambohitsitondroensis"
35,"Pseudophygasia apolinaria"
35,"Pseudophygasia furax "

MP-LI	Mpumalanga-Limpopo	13

48,"Afroaltica parvula"
48,"Calotheca luteotessellata"
48,"Chaetocnema mapumalangaensis "
48,"Chaetocnema sp n 4"
48,"Chaetocnema sp n 7"
48,"Chaetocnema sp n 9"
48,"Longitarsus transvaalensis"
48,"Serraphula alticola"
48,"Serraphula duplessisi"
48,"Serraphula grobbelaariae"
48,"Serraphula mpumalangaensis"
48,"Serraphula transvaalensis"
48,"Serraphula wittmeri"

DKM-KWN	Drakensberg Mountains-KwaZulu Natal	17

52,"Chaetocnema barkeri"
52,"Chaetocnema longicornis"
52,"Chaetocnema mariobiondii"
52,"Chaetocnema sp n 5"
52,"Chaetocnema sp n 6"
52,"Chaetocnema zulu"
52,"Drakensbergianella rudebecki"
52,"Jacobyana sudafricana"
52,"Longitarsus grobbelaariae"
52,"Serraphula audisiana"
52,"Serraphula burlischi"
52,"Serraphula colonnellii"
52,"Serraphula drakensbergensis"
52,"Serraphula monticola"
52,"Serraphula natalensis"
52,"Serraphula osellai"
52,"Serraphula puncticollis"

WCP Western Cape Province 31

56,"Adamastoraltica humicola"
56,"Calotheca danielssoni"
56,"Calotheca regularis"
56,"Chaetocnema adamastori"
56,"Chaetocnema audisiana"
56,"Chaetocnema chalcea"
56,"Chaetocnema convexicollis"
56,"Chaetocnema saldanhai"
56,"Chaetocnema sp n 1"
56,"Chaetocnema tablensis"
56,"Chirodica cedarbergensis"
56,"Chirodica chalcoptera unicolor"
56,"Chirodica fulvipes"
56,"Chirodica similfulva"
56,"Longitarsus afromeridionalis"
56,"Longitarsus capensis"
56,"Longitarsus cedarbergensis"
56,"Longitarsus debiasei"
56,"Longitarsus hexrivierbergensis"
56,"Longitarsus luctuosus"
56,"Longitarsus lugubris"
56,"Longitarsus malherbei"
56,"Longitarsus melanicus"
56,"Longitarsus neseri"
56,"Longitarsus piketbergensis"
56,"Longitarsus rouxi"
56,"Longitarsus sudafricanus"
56,"Stegnaspea audisiana"
56,"Stegnaspea danielssoni"
56,"Stegnaspea endroedyi"
56,"Stegnaspea penrithae"

Class 3 : 200 km

KIL Kilimandjaro region 12

18,"Blepharidina kasigauensis"
18,"Blepharidina knighti"
18,"Blepharidina macarthuri"
18,"Blepharidina ornaticollis"
18,"Calotheca jacobyi"
18,"Chaetocnema sp n 10"
18,"Chaetocnema sp n 11"
18,"Collartaltica alluadi"
18,"Psylliodes afromontana"
18,"Psylliodes manobioides"
18,"Psylliodes montana"
18,"Psylliodes shirensis"

KLR Kivu Lake region 5

19,"Chaetocnema rutovuensis"
19,"Chaetocnema varicornis"
19,"Guilielmia leleupi"
19,"Guilielmia monticola"
19,"Jacobyana centrafricana"

KAR Katanga region 7

28,"Angulaphthona confusa"
28,"Chaetocnema kapirensis"
28,"Chaetocnema muya"
28,"Chaetocnema reprehensa"
28,"Chaetocnema sp n 2"
28,"Chaetocnema sp n 3"
28,"Collartaltica nigrolucens"

AMM+BER+ANR Amber Mountain-Betsiboka region-Antananarivo region 51

31,"Chabria betsilea"
31,"Chabria betsimisaraka"
31,"Chabria bezanozana"
31,"Chaetocnema basipunctata"
31,"Chaetocnema cachani"
31,"Chaetocnema coronilla"
31,"Chaetocnema madascariensis"
31,"Chaetocnema orophila"
31,"Chaetocnema picipes"
31,"Diphaulacosoma bicolor "
31,"Diphaulacosoma bicolor"
31,"Diphaulacosoma crassicornis"
31,"Diphaulacosoma elegans"
31,"Diphaulacosoma jenisi"
31,"Diphaulacosoma nigroscutis"
31,"Neodera amplicollis"
31,"Neodera bernieri"
31,"Neodera breviantennata"
31,"Neodera didiensis"
31,"Neodera difficilis"
31,"Neodera diversitarsis"
31,"Neodera emarginata"
31,"Neodera fulva"
31,"Neodera imitatrix"
31,"Neodera jenisi"
31,"Neodera kraussi"
31,"Neodera longicollis"
31,"Neodera madagassa"
31,"Neodera marojejyensis"
31,"Neodera micheli"
31,"Neodera nigrotibialis"
31,"Neodera opaca"
31,"Neodera pauliani"
31,"Neodera perroti"
31,"Neodera peyrierasi"
31,"Neodera picticornis"
31,"Neodera reducta"
31,"Neodera similvadoni"
31,"Neodera sogai"
31,"Neodera straminea"
31,"Neodera straminoides"
31,"Neodera transversicollis"
31,"Neodera vadoni"
31,"Neodera varicornis"
31,"Ntaolaltica antennata"
31,"Pseudophygasia ambohitsitondroensis"
31,"Pseudophygasia analis"
31,"Pseudophygasia apolinaria"
31,"Pseudophygasia denotata"
31,"Pseudophygasia freyi"
31,"Pseudophygasia furax "

MP-LI Mpumalanga-Limpopo 14

42,"Afroaltica parvula"
42,"Calotheca luteotessellata"
42,"Chaetocnema mapumalangaensis "
42,"Chaetocnema sp n 14"
42,"Chaetocnema sp n 4"
42,"Chaetocnema sp n 7"
42,"Chaetocnema sp n 9"
42,"Longitarsus transvaalensis"
42,"Serraphula alticola"
42,"Serraphula duplessisi"
42,"Serraphula grobbelaariae"
42,"Serraphula mpumalangaensis"
42,"Serraphula transvaalensis"
42,"Serraphula uysi"
42,"Serraphula wittmeri"

DKM-KWN Drakensberg Mountains-KwaZulu-Natal 19

45,"Calotheca wanati"
45,"Chaetocnema barkeri"
45,"Chaetocnema longicornis"
45,"Chaetocnema mariobiondii"
45,"Chaetocnema sp n 5"
45,"Chaetocnema sp n 6"
45,"Chaetocnema zulu"
45,"Drakensbergianella rudebecki"
45,"Jacobyana sudafricana"
45,"Longitarsus grobbelaariae"
45,"Serraphula audisiana"
45,"Serraphula burlischi"
45,"Serraphula colonnellii"
45,"Serraphula drakensbergensis"
45,"Serraphula monticola"
45,"Serraphula natalensis"
45,"Serraphula osellai"
45,"Serraphula puncticollis"
45,“Calotheca carolineae”

WCP	Western Cape Province	32

49,"Adamastoraltica humicola"
49,"Calotheca danielssoni"
49,"Calotheca regularis"
49,"Chaetocnema adamastori"
49,"Chaetocnema audisiana"
49,"Chaetocnema chalcea"
49,"Chaetocnema convexicollis"
49,"Chaetocnema saldanhai"
49,"Chaetocnema sp n 1"
49,"Chaetocnema tablensis"
49,"Chirodica cedarbergensis"
49,"Chirodica chalcoptera unicolor"
49,"Chirodica fulvipes"
49,"Chirodica similfulva"
49,"Longitarsus afromeridionalis"
49,"Longitarsus capensis"
49,"Longitarsus cedarbergensis"
49,"Longitarsus debiasei"
49,"Longitarsus hexrivierbergensis"
49,"Longitarsus luctuosus"
49,"Longitarsus lugubris"
49,"Longitarsus malherbei"
49,"Longitarsus melanicus"
49,"Longitarsus neseri"
49,"Longitarsus piketbergensis"
49,"Longitarsus rouxi"
49,"Longitarsus sudafricanus"
49,"Stegnaspea audisiana"
49,"Stegnaspea danielssoni"
49,"Stegnaspea debiasei"
49,"Stegnaspea endroedyi"
49,"Stegnaspea penrithae"
